# Stabilizing spin systems via symmetrically tailored RKKY interactions

**DOI:** 10.1038/s41467-019-10516-2

**Published:** 2019-06-12

**Authors:** Jan Hermenau, Sascha Brinker, Marco Marciani, Manuel Steinbrecher, Manuel dos Santos Dias, Roland Wiesendanger, Samir Lounis, Jens Wiebe

**Affiliations:** 1 Department of Physics, Hamburg University, 20355 Hamburg, Germany; 2 Peter Grünberg Institut and Institute for Advanced Simulation, Forschungszentrum Jülich & JARA, 52425 Jülich, Germany; 3 Department of Physics, RWTH Aachen University, 52056 Aachen, Germany; 4 Instituut-Lorentz, Universiteit Leiden, P.O. Box 9506, 2300 RA Leiden, The Netherlands; 5 Present Address: Univ Lyon, Ens de Lyon, Univ Claude Bernard, CNRS, Laboratoire de Physique, 69342 Lyon, France

**Keywords:** Magnetic properties and materials, Quantum information, Spintronics

## Abstract

Spins of single atoms adsorbed on substrates are promising building blocks for spintronics and quantum computation schemes. To process spin information and for increased magnetic stability, these spins have to be coupled to arrays. For a single atom, a high symmetry of the environment increases its spin stability. However, little is known about the role of the symmetry of the magnetic couplings in the arrays. Here, we study arrays of atomic spins coupled via Ruderman−Kittel−Kasuya−Yosida interaction, focusing on Dzyaloshinskii−Moriya and symmetric anisotropic exchange. We show that the high spin stability of a trimer can be remotely detected by a nearby atom, and how the Dzyaloshinskii−Moriya interaction leads to its destabilization. Adding more nearby atoms further destabilizes the trimer, due to a non-local effective transverse anisotropy originating in the symmetric anisotropic exchange. This transverse anisotropy can be quenched for highly symmetric structures, where the spin lifetime of the array increases drastically.

## Abstract (web summary)

Exploration of the atomic spin interactions promises next generation information technologies. Here the authors show the observation and understanding of the Dzyaloshinskii−Moriya and symmetric anisotropic exchange interactions controlled spin dynamics and stability in Fe cluster-adatom complexes on Pt surfaces.

## Introduction

In magnetic systems, the enhancement, preservation or breaking of symmetries have strong implications for the ground-state and the dynamical properties. For instance, two-dimensional spin lattices with C_4V_ symmetry and antiferromagnetic nearest-neighbor interactions form a stable diagonal row-wise antiferromagnet, while a C_6V_ symmetry is predicted to have a spin liquid ground state^1–3^. The breaking of inversion symmetry is responsible for the presence of the Dzyaloshinskii−Moriya (DM) interaction between the spins in materials with large spin−orbit interaction^4,5^, which promotes the formation of magnetic skyrmions^6–9^. Another important example is the symmetry of the Coulomb field of the atoms around the spin, the so-called crystal field, which induces a magnetic anisotropy energy that leads to the stabilization of the spin along particular directions^10^. It has recently been shown that, when the crystal field of a single absorbed atomic spin has high symmetry^11^, and the spin is decoupled from the conduction electrons of the substrate, the spin state lifetime can be enormously enhanced, up to hours^12^. This brings the use of such atomic spins as bits of information technology into reach.

In order to read, write and process the information stored in the state of such atomic spins, they have to be coupled to neighboring spins. The transfer of information can be realized via dipolar interaction^13^, direct or superexchange interaction^14^, or the conduction-electron-mediated Ruderman−Kittel−Kasuya−Yosida (RKKY) interaction between spins on metallic surfaces^15–17^. The latter is unique in its flexibility to tailor the properties of the spin-system in terms of coupling strength and noncollinearity^18^. The general capability of RKKY-coupled networks to transfer and process information at the nanoscale has been shown by realizing few-atom spin-based logic gates^19^. One might naively expect that enhancing the number of atoms in such a network will drive the system into the regime of a classical magnet, where spin-fluctuations are less important. Indeed, exchange-coupling a few spins to larger ensembles via strong isotropic ferromagnetic (FM)^20^ or antiferromagnetic (AFM)^21,22^ Heisenberg exchange interactions has been shown to enhance the spin stability with respect to that of the constituents^23^. However, the coupling more generally has other contributions, such as the DM and the symmetric anisotropic Heisenberg exchange^24^. The effects of such contributions on the spin fluctuations and whether they are governed by symmetries remain to be explored. An experimental and theoretical approach towards this end for RKKY-coupled spins is given in the following, where the spin-fluctuations of atom-by-atom engineered complexes consisting of one cluster and a different number of RKKY-coupled satellite atoms are investigated. The results of time-resolved spin-polarized scanning tunneling microscopy experiments are analyzed and interpreted taking into account the outcome of density functional theory (DFT) calculations and dynamic simulations.

## Results

### Trimer–satellite atom complexes

**Fig. 1:**
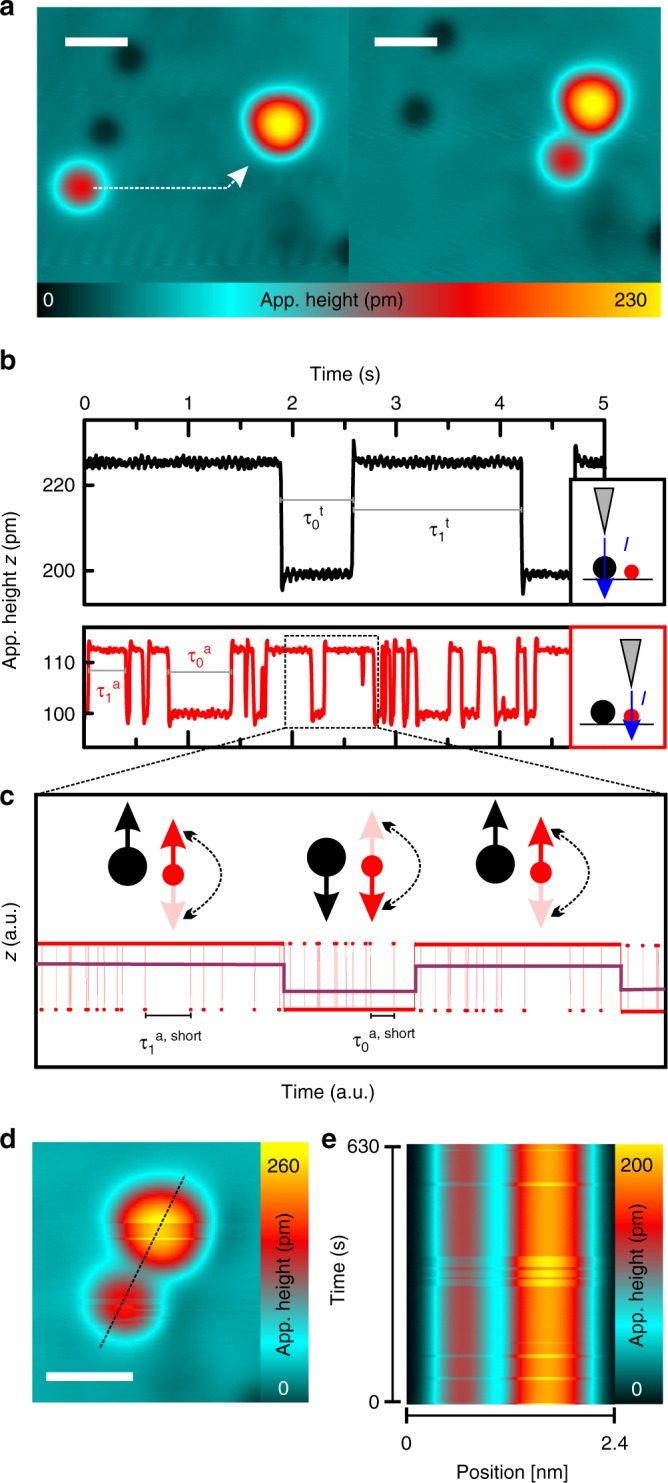
Assembly and spin switching of the trimer–satellite atom complex. **a** Constant-current STM images (5 × 5 nm^2^, the scale bars have a length of 1 nm, $V = 5\,{\mathrm{mV}}$, $I = 500\,{\mathrm{pA}}$) of the construction process of a complex via lateral atom manipulation ($V_{{\mathrm{manip}}} = 1.1\,{\mathrm{mV}}$, $I_{{\mathrm{manip}}} = 25\,{\mathrm{nA}}$). A single iron atom is approached to the trimer. Within the complex the atom sits on an fcc adsorption side labeled as *a* in Fig. 2a. In this complex, the distance between atom and trimer is *d* = 0.97 nm. **b** Time traces of the apparent height recorded on the trimer (black, upper panel) and on the atom (red, lower panel) (*V* = 2.75 mV, *I* = 200 pA, *B* = 0 T, *T* = 300 mK). The data sets are shifted vertically for better visibility. **c** Illustration of the contrast mechanism for the data taken on the fast switching atom ferromagnetically coupled to a trimer in an SP-STM measurement. The fast switching events are averaged out during the measuring time, leading to a change in the apparent height of the atom when the trimer switches as indicated by the dark red lines. **d** Constant-current STM image of the complex recorded with an out-of-plane spin-polarized tip (the scale bar has a length of 1 nm, *V* = 3 mV, *I* = 50 pA, *B* = 0 T, *T* = 300 mK). **e** Consecutively recorded spin-resolved line scans along the dashed line in (**d**) stacked on top of each other (*V* = 3 mV, *I* = 20 pA, *B* = 0 T, *T* = 300 mK, scanning time per line = 614 ms)

A prototypical system for these studies consists of iron (Fe) atoms on a Pt(111) surface (Fig. 1a, see Methods), which adsorb either on the face-centered cubic (fcc) or on the hexagonal close-packed (hcp) sites. In both cases, the atoms can be approximately described by an effective spin with a quantum number of *S*_a_ = 5/2, and a preferred spin orientation along the surface normal (fcc, easy-axis anisotropy) or in the surface plane (hcp, easy-plane anisotropy)^25^. Up to distances of several lattice spacings, there is a sizeable RKKY interaction between the Fe atoms with significant Heisenberg as well as DM contributions. Varying the separation of the spins gives control over the magnitudes and signs of the Heisenberg and DM parts, in order to tailor noncollinear spin states^18,26^. Contrary to what one may expect intuitively, the strong interaction of Fe atoms with the Pt(111) substrate does not prevent ground state spin lifetimes of hours, when at least three iron atoms are manipulated into a close-packed fcc-top-stacked symmetrical trimer (Fig. 1a) with a spin of *S*_t_ = 11/2^27^. Since the spin state of these trimers (up or down, perpendicular to the surface) can be easily set by a short pulse of spin-polarized electrons with sufficient energy, they present ideal inputs/switches for RKKY-coupled networks of atoms^19^. However, it is a priori unclear whether the RKKY interaction, e.g. between such a trimer and a close-by satellite atom (Fig. 1a), further stabilizes or destabilizes the spin state of the coupled network. In order to investigate this question, we first place a single Fe atom on an fcc site at a distance of *d* = 0.97 nm to a well-characterized fcc top trimer^27^, via lateral atom manipulation (Fig. 1a).

### Two spin-state telegraph noise

The strong impact of the RKKY coupling on the dynamics of the trimer−satellite atom system becomes apparent when a magnetic tip (sensitive to the out-of-plane component of the sample spin-polarization) is positioned on either of the two constituents of this complex (Fig. 1b). For both tip positions, we observe a telegraph noise between two spin states, 0 and 1, with corresponding lifetimes of *τ*_0_ and *τ*_1_ and mean lifetime $\tau = (\tau _0^{ - 1} + \tau _1^{ - 1})^{ - 1}$ on the order of 10 ms to 10 s. Similar spin-state lifetimes have been observed for isolated uncoupled trimers and are associated with the tunneling electron-induced dynamics between two degenerate out-of-plane oriented ground states of the trimer^27^. However, the observation of a lifetime on this order of magnitude for the spin state of the satellite atom is surprising. For isolated uncoupled atoms, the spin ground state lifetimes have been estimated to be on the order of 1 ms^28^ prohibiting the observation of the corresponding telegraph noise due to the limited time resolution of the method. Therefore, the telegraph noise detected on the satellite atom (Fig. 1b bottom trace) must be a consequence of its RKKY interaction with the slowly switching trimer. We propose the mechanism illustrated in Fig. 1c, which has been confirmed by our simulations (see below and Supplementary Notes 6 and 7). The RKKY interaction with the trimer induces an asymmetry $\frac{{\tau _{\mathrm{1}}^{{\mathrm{a}},{\mathrm{short}}} - \tau _0^{{\mathrm{a}},{\mathrm{short}}}}}{{\tau _1^{{\mathrm{a}},{\mathrm{short}}} + \tau _0^{{\mathrm{a}},{\mathrm{short}}}}}$ in the short spin-ground-state lifetimes of the satellite atom ($\tau _{0/1}^{{\mathrm{a}},{\mathrm{short}}}$). Although the SP-STM measurement averages out this fast telegraph noise, a changing asymmetry induced by switches of the trimer spin leads to a changing apparent height *z* of the satellite atom, which is detectable via the longer lifetimes $\tau _0^{\mathrm{a}}$ and $\tau _1^{\mathrm{a}}$. Thereby, the telegraph noise which is measured on the satellite atom reflects only the slow dynamics of the coupled system and essentially enables a nonlocal read-out of the trimer spin similar to ref. ^21^. This interpretation is further corroborated by the correlation between the telegraph noises measured on the trimer and satellite atom (Fig. 1d, e). In consecutively recorded line scans along the dashed line in Fig. 1d, which are stacked on top of each other in Fig. 1e, an anticorrelated switching of the trimer and satellite atom is found. Whenever the trimer is in the high state, the apparent height of the atom is low, and vice versa. Such anticorrelations or correlations of the two telegraph signals furthermore enable to conclude on a dominating AFM or FM orientation between the trimer and the satellite atom, since atom^29^ and trimer possess the same sign of the vacuum spin-polarization (Supplementary Note 4). The anticorrelation observed in Fig. 1e therefore indicates an AFM spin orientation in this particular complex.

### Dependence of lifetime on trimer to satellite-atom distance

Although the telegraph noises measured in the two situations depicted in the insets in Fig. 1b, i.e. directly on the trimer (black) or nonlocally on the satellite atom (red), reflect the dynamics of the coupled system, they strongly differ for the two cases. Consequently, these differences must originate from different probabilities to switch the trimer–satellite atom complex for the two tunneling paths. Interestingly, for the particular complex in Fig. 1, the tunnel current is more likely to induce switching via the atom than via the trimer ($\tau _{\mathrm{a}} < \tau _{\mathrm{t}}$), and both lifetimes are smaller than the lifetime measured on the same trimer in the absence of the satellite atom^27^. Counter-intuitively, the trimer spin is found to be destabilized by the nearby atom.

**Fig. 2:**
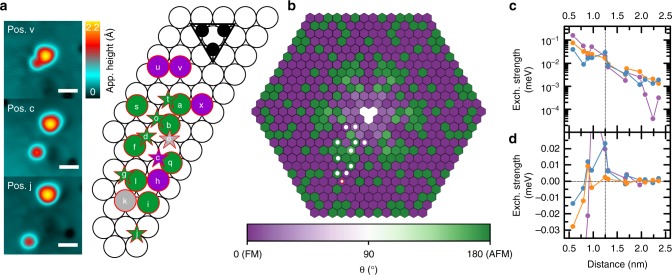
Distance dependence of the spin configuration and the coupling strength. **a** Experimentally investigated atom positions labeled by the chronological order were addressed in the experiment (the scale bars have a length of 1 nm). Fcc (hcp) positions are marked with circles (stars). The color depicts whether a FM (purple) or an AFM (green) switching was revealed experimentally as the one shown for position a in Fig. 1d, e. For the positions shown in gray, no contrast/switching on the atom was found. Three exemplary complex configurations are shown in the constant-current images on the left. **b** Ground state spin configurations for fcc adsorbed satellite atoms determined from a classical Heisenberg model using parameters obtained from DFT calculations. The trimer atoms sit on the three fcc sites in the center (white). $\theta$ describes the angle between the atom and the trimer spin. The fcc positions addressed in the experiment are marked by circles (the position which shows a mismatch between experiment and DFT is given in red). **c** Strengths |*J*_iso_| (purple), |**J**_aniso_| (orange) and |**D**| (blue) in dependence of the distance between the trimer and the atom (see Supplementary Note 8 for the definition of the strengths). **d** *J*_iso_ (purple), *J*_*xx*_ (orange) and *D*_*x*_ (blue) in dependence of the trimer–atom separation

In order to systematically study the influence of the satellite atom on the magnetization dynamics of the complex, various structures are constructed and characterized in terms of dynamics and coupling behavior. To this end, the satellite atom is moved to adsorption sites at various distances to the trimer via lateral atom manipulation (Fig. 2a). In all these complexes, a finite coupling was revealed by a telegraph noise on the satellite atom analogous to the observations for position a in Fig. 1. In dependence of the actual position of the satellite atom, the telegraph noises of trimer and atom are correlated or anticorrelated, indicating a complex oscillation between FM and AFM orientation illustrated in Fig. 2a by the corresponding color. This oscillatory behavior is reminiscent of an RKKY interaction. Indeed, the measured orientations are consistent with the configurations that have been obtained from the minimization of a classical Heisenberg model using density DFT parameters as input (Fig. 2b, see Methods). The DFT calculations were performed for all fcc positions of the satellite atoms under the assumption of a collinear internal spin configuration in the out-of-plane direction for the trimer, which is a valid approximation for fcc trimers due to only tiny noncollinear deviations^27^. The calculations reveal that the magnetic moment of the satellite atom is almost collinear to that of the trimer (along the *z*-axis, which is the easy axis for fcc atoms). Only for the farthest separation between trimer and satellite atom are the experimental and theoretical results in contradiction, which might be due to inaccuracies in the calculation associated with the small size of the interaction. The FM-AFM oscillation results from a complex interplay of different contributions to the RKKY interaction, as revealed by mapping the DFT calculated energies to a generalized classical Heisenberg model which then results in the quantum Heisenberg Hamiltonian

1 $$\hat H = J_{{\mathrm{iso}}}{\hat{\mathbf{S}}}_{\mathrm{a}} \cdot {\hat{\mathbf{S}}}_{\mathrm{t}} + {\hat{\mathbf{S}}}_{\mathrm{a}} \cdot \underline {\mathbf{J}} _{{\mathrm{aniso}}} \cdot {\hat{\mathbf{S}}}_{\mathrm{t}} + {\mathbf{D}} \cdot {\hat{\mathbf{S}}}_{\mathrm{a}} \times {\hat{\mathbf{S}}}_{\mathrm{t}} + {\cal{D}}_{\mathrm{a}}\left( {\hat S_{\mathrm{a}}^z} \right)^2 + {\cal{D}}_{\mathrm{t}}\left( {\hat S_{\mathrm{t}}^z} \right)^2,$$

with the vector spin operators of the satellite atom ${\hat{\mathbf{S}}}_{\mathrm{a}}$ and trimer ${\hat{\mathbf{S}}}_{\mathrm{t}}$ and their respective *z*-components $\hat S_{\mathrm{a}}^z$ and $\hat S_{\mathrm{t}}^z$. Besides the magneto-crystalline anisotropies (MAE) of atom (${\cal{D}}_{\mathrm{a}}$) and trimer (${\cal{D}}_{\mathrm{t}}$), it includes the most general form of Heisenberg exchange including the isotropic (*J*_iso_) and the symmetric anisotropic (**J**_aniso_) parts, as well as the DM interaction (**D**) (see Supplementary Notes 5, 6 for the definition of the operators and the relation between classical and quantum Heisenberg Hamiltonian). The symmetric anisotropic part, **J**_aniso_, is also called the pseudo-dipolar interaction or a two-ion anisotropy term, which contrary to the isotropic part, *J*_iso_, favors the orientation of the moments along specific directions similarly to MAE, leading to nonlocal effective magnetic anisotropies.

Figure 2c shows the distance-dependent strengths of *J*_iso_, **J**_aniso_ and **D**, whose components all display oscillations in the sign typical for RKKY interaction (see Fig. 2d). These sign oscillations, together with a rapid decrease in the strength of the RKKY interaction (Fig. 2c) and the relatively strong out-of-plane MAE of the fcc satellite atoms ${\cal{D}}_{\mathrm{a}} = - 0.19\,{\mathrm{meV}}$^25^ and trimer ${\cal{D}}_{\mathrm{t}} = - 0.09\,{\mathrm{meV}}$^27^ lead to an almost collinear spin orientation between trimer and satellite atom along the surface normal, which alternates between FM and AFM as their separation is varied. For complexes including hcp satellites, the experimental results (Supplementary Note 1) point towards a deviation of the atom’s spin orientation from the surface normal due to the in-plane MAE.

**Fig. 3:**
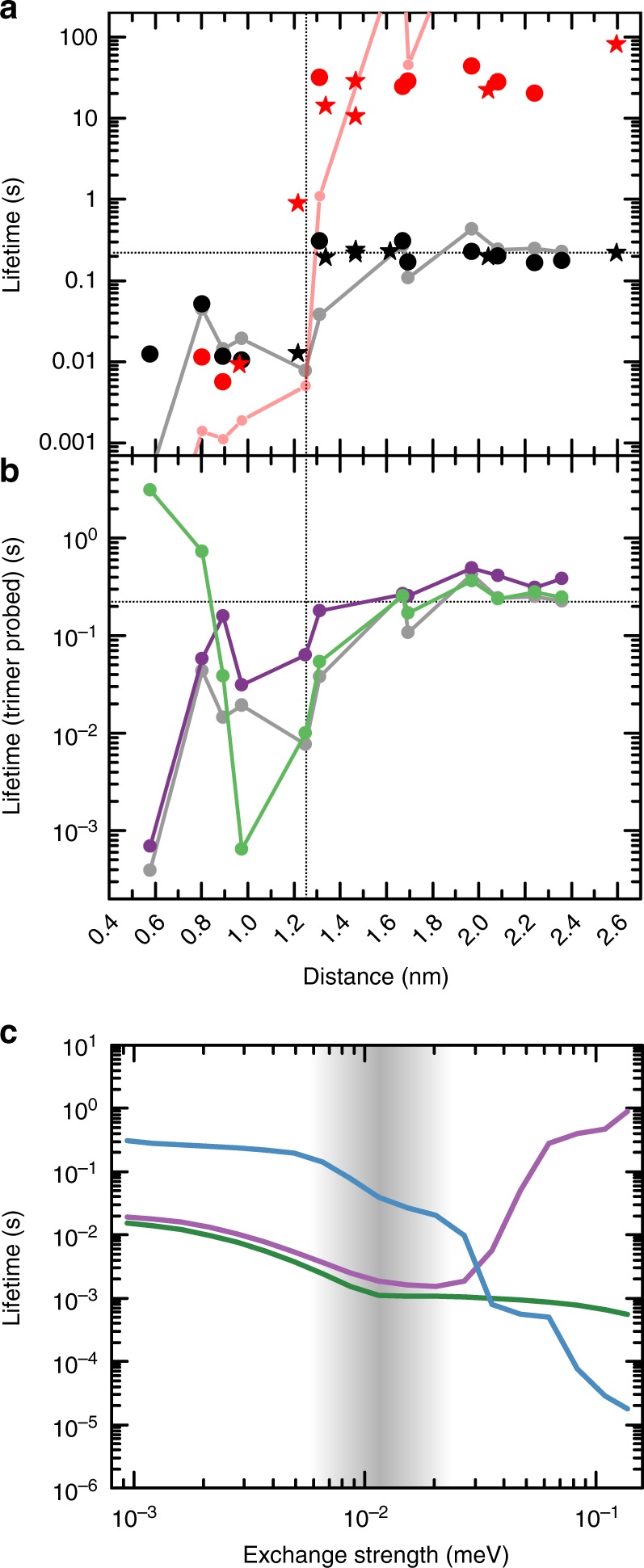
Distance dependence of the spin lifetime. **a** Mean lifetimes of the data recorded on the trimer $\tau _{\mathrm{t}}$ (black) and on the atom $\tau _{\mathrm{a}}$ (red) in dependence of the trimer−atom separation (Note, that the lifetimes for the separation corresponding to the configuration in Fig. 1a differ from those in Fig. 1b due to a different measurement voltage, *V* = −5 mV, *I* = 500 pA, *B* = 0 T and *T* = 300 mK). Data points of the complexes with an fcc (hcp) adsorbed satellite atom are marked with a circle (star). The horizontal (vertical) dashed line marks the lifetime of the free-standing trimer (the crossover between the two lifetime regimes). In gray (weak red) the simulated lifetimes for the scenario of the tip probing the trimer (the satellite atom) are given. **b** Calculated lifetimes probed on the trimer for the experimental separations using different parts of the exchange interactions. The reference data using the full exchange interactions are shown in gray. The lifetimes calculated without the symmetric anisotropic exchange are shown in green. The lifetimes calculated without the DM interaction are shown in purple. **c** Calculated lifetimes probed on the trimer in dependence of the isotropic exchange interaction *J*_iso_ (AFM: green, FM: purple) and the DM interaction *D*_*x*_ (blue). For the calculations, the two remaining exchange contributions are set to zero (Supplementary Note 7). The gray area marks the regime of the exchange constants found for an atom−trimer separation of about 1.25 nm

**Fig. 4:**
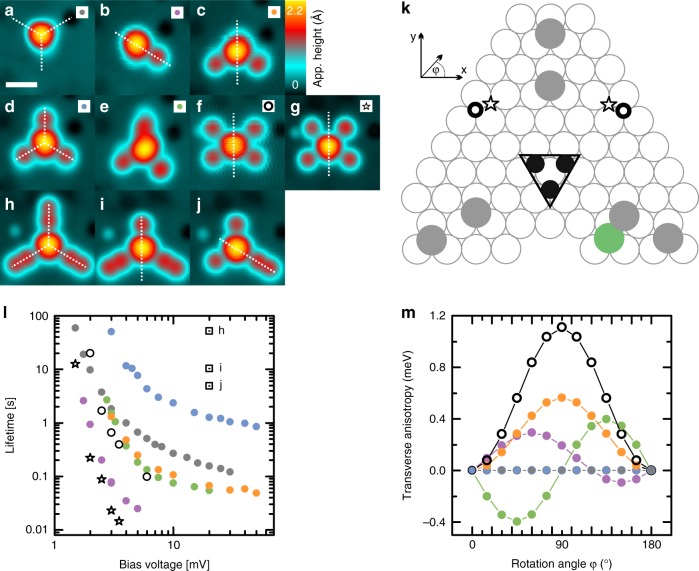
Magnetic stability of multisatellite complexes. **a**–**j** Constant current images of the investigated complexes (the scale bar has a length of 1 nm). For (**b**–**d**) the distance between satellite atom and closest trimer atom is *d* = 0.8 nm. **k** The corresponding adsorption sites of the complexes shown in (**a**–**j**). **l** Bias dependence of the lifetimes measured on the trimer of the structures marked in (**a**–**j**) (*I* = 750 pA *B* = 0 T and *T* = 300 mK, colors/symbols are given in **a**–**j**). **m** Effective magnetic anisotropy energy of the spin system in dependence of a collinear rotation of the trimer and adatom spins in the sample surface (colors/symbols are given in **a**–**j**, 0° parallel to *x*-axis with the coordinates indicated in **k**)

In order to investigate the influence of these RKKY couplings on the dynamics of the complexes, time traces like those in Fig. 1b were recorded for each built configuration. The lifetimes $\tau _{\mathrm{a}}$ and $\tau _{\mathrm{t}}$ measured with the tip on the satellite atom, respectively the trimer, are plotted in Fig. 3a against the atom-trimer separation. Obviously, there are two qualitatively different regimes. For smaller distances ($d < 1.25\,{\mathrm{nm}}$, see vertical dashed line), there is a destabilization of the trimer by the satellite atom in comparison to the free-standing case (horizontal dashed line). Independently of whether the complex lifetime is measured via the atom (red) or directly on the trimer (black), the values are about one order of magnitude smaller. In contrast, for larger distances ($d > 1.25\,{\mathrm{nm}}$), the lifetime of the complex strongly differs between the two measurement modes: When the current runs through the trimer ($\tau _{\mathrm{t}}$) the lifetime is comparable to that of an isolated trimer, which indicates a negligible influence of the satellite atom on the trimer dynamics. However, the two-state telegraph noise can still be observed if the current runs through the satellite atom ($\tau _{\mathrm{a}}$), proving a finite RKKY coupling between the two constituents. The accordingly-measured values of $\tau _{\mathrm{a}}$ are two orders of magnitude larger than $\tau _{\mathrm{t}}$, indicating that the influence of the measurement process on the trimer dynamics is drastically reduced. No clear correlation is found between the lifetime and the type of the adsorption site of the satellite atom nor the type of coupling, AFM or FM. Also, the destabilization effect in the small distance regime is independent of the used bias voltage, as it becomes obvious by comparison of the data from the free-standing trimer and the complex recorded over a large range of bias voltages (Fig. 4l).

To unravel the physics behind the sharp crossover between the two regimes, we performed simulations within a master equation approach, in conjunction with the effective quantum spin Hamiltonian given above^20,27^ using the exchange parameters extracted from the DFT calculations (see Methods). In both regimes, the simulations reproduce the experimental data quantitatively (Fig. 3a). Only for larger distances and when the current flows through the satellite atom, does the model overestimate the lifetimes, which might be explained by quantum fluctuations due to additional transversal magnetic anisotropies^28^ or zero-point spin fluctuations^30^ which go beyond those included in the master equation approach. Note, that an effective tip-magnetic field^27,28^ may have residual effects on the lifetime difference between the measurements with the tip above of the trimer or above the satellite atom, which are not considered in the simulations. However, the effective field of the particular tip used for the measurements of Fig. 3a has been estimated to be smaller than 0.17 T which results in negligible changes of the lifetimes by at most a factor of 2 (Supplementary Notes 2 and 3). Moreover, an analysis using the magnetic-field-independent geometric means for the determination of the lifetimes instead of the harmonic means used for Fig. 3a, and additional data measured on another set of manipulated complexes using another tip, both show quantitatively similar results (Supplementary Note 3), ruling out a major role of the tip-effective magnetic field for the observed distance dependence of the lifetime.

The simulations enable to identify the origin of the steep increase of the lifetimes around 1.25 nm by independently sweeping D_*x*_ and *J*_iso_ (Fig. 3c). In the experimentally relevant parameter regime of atom−trimer separations of around 1.25 nm, there is a strong drop of the lifetime when the DM interaction strength exceeds ≈6 × 10^−3^ meV. The effect of the isotropic exchange interaction on the lifetime is much less pronounced. This strongly points towards a dominant destabilization of the trimer spin by the DM interaction. Further studies separating the dependence of the lifetime on the three different exchange contributions reinforce this finding (Fig. 3b). Contrary to the DM interaction, the symmetric anisotropic exchange interaction can affect the lifetimes in two ways: It can either stabilize the complex by inducing an effective easy-axis anisotropy (e.g. via a ferromagnetic *zz*-component) or destabilize it, similarly to the DM interaction, by inducing an effective transversal anisotropy. Figure 3b shows that the destabilization of the lifetime for an atom−trimer separation of around 1.25 nm is mainly driven by the DM interaction, whereas in the close distance regime the symmetric anisotropic part of the exchange interaction dominates the destabilization.

### Dependence of the dynamics on symmetry

The observed destabilization of the spin-structure by coupling the trimer to a satellite atom is rather counterintuitive, as generally systems tend to stabilize with an increasing number of spins^20,22^. Importantly, the addition of the satellite atom not only increases the number of spins, but also lowers the symmetry of the system. These two effects on the lifetime, i.e. number of spins and symmetry, are investigated in the following by adding different numbers of satellite atoms in different configurations to the complex (Fig. 4a–l). The lifetime depends in a rather intricate manner on the number of satellite atoms. It first increases for two satellite atoms (Fig. 4c, l), but then stays almost the same for three (Fig. 4e) and four (Fig. 4f) satellite atoms, or is even drastically reduced for a different position of the four satellite atoms (Fig. 4g). Note, that from (Fig. 4b) to (Fig. 4g) all satellite atoms have a distance *d* < 1.25 nm to the trimer, i.e. within the regime of strong coupling (cf. Fig. 3a). In most of these complexes the lifetime is smaller than for the symmetrical case of the trimer without any satellite atoms (Fig. 4a). Only if the satellite atoms are added in a way that conserves the C_3V_ symmetry of the trimer/substrate (Fig. 4d), can the lifetime be further increased by an order of magnitude above the one of the isolated trimer.

This suggests that keeping a high symmetry of the RKKY interactions, and in particular of the symmetric anisotropic contribution which dominates the destabilization in the complex with one satellite atom in the close distance regime, restores the spin-stability. The effect of the symmetry of the RKKY interactions on the lifetime can be rationalized by the DFT calculation of the effective magnetic anisotropy energy of all trimer–satellite atom complexes when the spins of all constituents are collinearly rotated in the surface plane (Fig. 4m). Interestingly, the RKKY interaction induces an effective nonlocal transversal magnetic anisotropy in the complex spin system, whenever the C_3V_ symmetry is broken resulting in C_S_ or no symmetry. The crucial effect of the symmetry on this nonlocal transversal magnetic anisotropy and thereby on the lifetime of the complex is further proven by simulations of slight distortions of the symmetric complex of Fig. 4d (Supplementary Note 7 and Supplementary Fig. 10). Already a slight misalignment of one of the satellite atoms from the symmetric position decreases the lifetime by an order of magnitude, although the anisotropies and exchange couplings are kept constant. On-site transversal magnetic anisotropy in single-spin effective Hamiltonians is well known to induce instability of the lifetime via mixing of the spin states^31,32^. However, the related effect in a multispin complex originating from the nonlocal RKKY interactions between the spins, which we observe here, has not been studied so far, and cannot be reduced to the single-spin effective Hamiltonian picture.

We consequently expect that it might be possible to further stabilize the complex by adding a larger number of satellite atoms in symmetrical positions. This idea is experimentally verified by the formation of a symmetrical complex with six satellites shown in Fig. 4h. Successively adding three more atoms on symmetrical positions to the complex in (Fig. 4d), the lifetime is further increased by more than an order of magnitude (see the intermediate steps in (Fig. 4i, j) and the corresponding lifetimes in Fig. 4l).

## Discussion

In conclusion, we revealed the dynamic behavior of RKKY-coupled complexes of two and more constituents. There are three regimes depending on the coupling strength between the trimer and a single satellite atom: In the weak coupling regime, the nearly intrinsic dynamics of the trimer could be observed while probing the satellite atom. This enables to nonlocally read and write the trimer spin via an RKKY-coupled network of atoms. In the intermediate coupling regime, the DM interaction leads to a significant destabilization when its magnitude exceeds a certain threshold. In the strong coupling regime, the symmetric anisotropic exchange interaction leads to a nonlocal transverse effective magnetic anisotropy which generally destabilizes the system. However, in the latter case, by engineering the symmetry of the complex, the magnetic stability can be restored and even enhanced by an order of magnitude. This demonstrates an interesting direction for the tailoring of RKKY-coupled networks of atoms towards usage in spintronics elements.

## Methods

### Experimental procedures

All measurements have been performed under ultra-high vacuum conditions in a home-built scanning tunneling microscope facility at a base temperature of *T* = 300 mK. A superconducting magnet enabled to apply a magnetic field *B* perpendicular to the sample surface^33^. The Pt(111) single crystal was cleaned in situ by argon ion sputtering and annealing cycles and a final flash, and single Fe atoms were subsequently deposited onto the cold substrate, as described in ref. ^25^. The experiments described here have been conducted using the same preparation as used for the measurements presented in refs ^26–28^.

The trimer was constructed by lateral atom manipulation^34^ in the pulling mode. The construction and identification is described in detail in ref. ^27^. With the same technique, the arrangement of satellite atoms in the complexes is changed. During such a rearrangement of the complex, residual vertical or lateral drift is compensated. This enables to exclude tip changes by comparing the absolute substrate height before and after each manipulation. The stacking of the new position of the satellite atom is revealed by inelastic scanning tunneling spectroscopy^25^. The trimer is not moved within the presented experiments.

All measurements presented here are performed using a spin-polarized tip with a magnetically stable sensitivity to the out-of-plane component of the sample magnetization. To this end, the spin sensitivity of a Cr-coated tungsten tip was optimized by picking up single iron atoms until a high two-state telegraph noise is observed on the trimer.

Constant-current images were taken using a tunneling current *I* with a bias voltage *V* applied to the sample. For the dynamic measurements, the tip is placed stationary above the atom or the trimer and the apparent height is recorded with running feedback loop stabilizing the current at *I*.

### Ab initio calculations

For the ab initio DFT calculations, we employed the Korringa−Kohn−Rostoker Green function method in full potential with spin−orbit coupling added to the scalar-relativistic approximation^35^. The exchange and correlation effects are treated in the local spin density approximation as parametrized by Vosko et al.^36^. The pristine Pt(111) surface is modeled by 40 Pt layers augmented by two vacuum regions. The magnetic Fe atoms are placed on the surface in the fcc-stacking position, using an embedding method. The magnetic anisotropy energies and the exchange interactions are obtained from band energy differences using the magnetic force theorem^37^ and the infinitesimal rotation method^37,38^, respectively. Both are used in an extended classical Heisenberg model to obtain the ground state spin configuration (see Supplementary Note 5). The DFT results for the spin moments of the iron atoms and their magnetic anisotropies and magnetic pair interactions were used to parametrize the master equation model.

### Master equation model

We use a master equation model to simulate the lifetime in the telegraph noise experiment. The magnetic clusters are described within the quantum Heisenberg model (Eq. 1) following refs ^20,27^. The interaction of the tunneling electrons with the spins is described by an Applebaum Hamiltonian^39^. The ratio between the coupling of the spins to the tip and to the surface at a bias voltage of 5 V is set to $\upsilon ^{\mathrm{T}}$/$\upsilon ^{\mathrm{S}} = 0.055$, as determined in a previous experiment^27^. The lifetimes are determined using a master equation approach combined with Fermi’s Golden rule^39^.

## Supplementary information

Supplementary Information

## Data Availability

The authors declare that the main data supporting the findings of this study are available within the article and its Supplementary Information files. Extra data are available from the corresponding author upon reasonable request.
