# Supplementary material for: Stabilizing spin systems via symmetrically tailored RKKY interactions
Source: Nat Commun. 2019 Jun 12;10:2565. doi: 10.1038/s41467-019-10516-2 (PMC6561942; doi:10.1038/s41467-019-10516-2)
Supplement: Supplementary file 1 — Supplementary Information [file 41467_2019_10516_MOESM1_ESM.pdf]

Stabilizing spin systems via symmetrically tailored RKKY interactions

Hermenau *et al.*

Supplementary Note 1 | Magnetic contrast on satellite atoms

We define the magnetic contrast Δz as the difference in the two apparent height levels in the corresponding telegraph signal. Except for the closest position addressed in the experiment, the contrast on the trimer is constant and independent of the voltage (Supplementary Figure 1). This not only proves the stability of the tip during the manipulation of the satellite atom but also strongly points towards a trimer spin pointing along the z-axis (perpendicular to the surface) in most of the built complexes. This observation justifies the fixing of the trimer spin in the DFT calculations discussed in Supplementary Note 4 |.

The contrast on the satellite atom is strongly dependent on the actual distance from the trimer but also on the used bias voltage. For the fcc adsorption sites (circles) a gradual fade out of the contrast towards larger atom-trimer separations is visible in Supplementary Figure 1. This observation is consistent with a rapid fluctuation of the atomic spin beyond the time resolution of SP-STM which is still influenced by the coupling to the trimer (see Fig.1c of the main text). In analogy to an effective magnetic field, this coupling induces an asymmetry in the population of the two magnetic ground states of the atom. In the relatively slow constant-current mode of a SP-STM measurement this switching can not be resolved but the measured effective apparent height depends on the time average of the apparent heights in the two magnetic ground states. Thus, in dependence on the spin state of the trimer a larger or a smaller effective apparent height is measured on the atom. Assuming a purely collinear alignment of atom and trimer magnetic moments (which is reasonable for the fcc satellites due to the large out-of-plane MAE[1]), a large contrast between these two heights hence depicts a strong difference of the asymmetry in the population of the two ground states caused by a strong coupling. On the other hand, the observed fade out of the contrast on the atom with increasing trimer-atom separation reflects the decreasing RKKY interaction of the trimer to the atom.

For hcp adsorbed atoms the behavior is similar, although the trend is less monotonous and there seem to be oscillations as a function of distance. This might be explained by a significant change of the canting of the atomic spin enabled by the much smaller MAE found for hcp atoms in comparison to fcc atoms[1]. Here the contrast is not only given by the strength of the overall coupling between adatom and trimer but also by the actual angle between atom spin and the tip's spin.

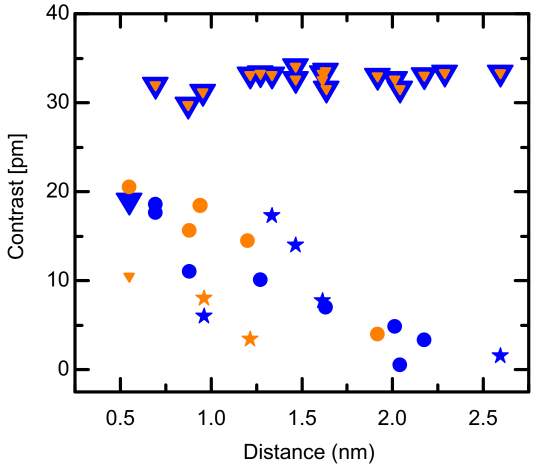

Supplementary Figure 1 | The magnetic contrast of the data in Fig.3a in the main text (blue data points) measured on the trimer (triangles) as well as on the satellites (fcc atom: circles, hcp atoms: stars) in dependence on the atom-trimer separation ($V = 5\,\mathrm{mV}$). Additionally, the contrast for telegraph signals recorded at $V = 3\,\mathrm{mV}$ is given for some distances in orange.

Supplementary Note 2 | Determination of the tip-effective magnetic field

In order to quantitatively extract the effective field of the tip used for the measurement in Fig. 3a of the main manuscript we analyzed the magnetic asymmetries of the telegraph signals of the complexes measured with the tip positioned on the trimer and satellite atom, as shown in Supplementary Fig. 2. The asymmetry is defined as

$$\mathcal{A} = \frac{\bar{\tau}_1}{\bar{\tau}_0 + \bar{\tau}_1} \tag{1}$$

with the time-averaged occupational lifetimes $\bar{\tau}_0 = \frac{1}{n}\sum_i \tau_{0,i}$ and $\bar{\tau}_1 = \frac{1}{n}\sum_i \tau_{1,i}$ in the two states 0 and 1. For each complex, the asymmetries are direct measures for the effective magnetic field acting on the trimer for the two measurement configurations with the tip above the trimer and the tip above the satellite atom. As seen in Fig. 1a of the main manuscript, the tip approaches by about $100\,\mathrm{pm}$ towards the surface for the measurement on the satellite atom with respect to the measurement on the trimer, which can potentially result in a slightly different effective field acting on the trimer for the two measurement positions. However, as shown in Supplementary Fig. 2a and b exemplarily for the complex in the configuration i, the asymmetries are rather similar, i.e. 0.64 on the trimer and $0.72 = 1 - 0.28$ on the satellite atom (coupled antiferromagnetically). This

indicates only a small change in the effective field acting on the trimer for the two measurement configurations. Moreover, the values do not strongly deviate from 0.5 generally indicating small effective fields of less than $0.2\,\mathrm{T}$ (c.p. Fig. 4 of Ref. 2 which shows that fields of more than $0.2\,\mathrm{T}$ result in asymmetries very close to 1 or 0). The asymmetries of the telegraph signals measured on trimer and satellite atoms for all complexes of Fig. 3a of the main manuscript are given in Supplementary Figs 2c. The absolute maximum and minimum values are 0.85 and 0.25, indicating rather small effective fields. The difference in asymmetries between the measurement on the trimer and satellite atom is always smaller than 0.35, which shows that this effective field acting on the trimer is also not strongly changing between the two measurement positions above trimer and above satellite atom. In order to determine the change in effective tip field from these asymmetries, we consider the magnetization curve of an individual trimer (without satellite atom) shown in Supplementary Fig. 2d. Using this curve, the above difference in absolute maximum and minimum values of the asymmetries translates into an upper border for the change of the tip effective field of only $\Delta B_{\text{max}} \approx 170\,\mathrm{mT}$. At least for the large distance range of the complexes (distances larger than $1.25\,\mathrm{nm}$) we can safely assume that this estimate remains valid for the complexes, as the very weakly coupled satellite atom will not strongly change the magnetization curve with respect to that shown in Supplementary Fig. 2d.

As visible in Supplementary Fig. 3a, this upper border of the effective-tip-field change results in an upper border of the change in the harmonic mean of the lifetime of the trimer in the complex between the two measurement configurations above trimer and above satellite atom of less than a factor of 2. The change in the lifetimes due to a residual tip effective field is thus by two orders of magnitudes too small to explain the observed changes in the lifetimes between the measurement on the trimer and on the satellite atom in the large distance regime of Fig. 3a of the main manuscript.

Supplementary Note 3 | Geometric means of the lifetimes and measurements with a different tip

The lifetimes shown in Fig. 3a of the main manuscript are calculated from the harmonic mean $\tau_{\text{harm}} = \left(\frac{1}{\bar{\tau}_0} + \frac{1}{\bar{\tau}_1}\right)^{-1}$. As shown by a magnetic-field dependent measurement on an individual trimer without satellite atom using a different tip from the one used for the data in the main manuscript, τ_{harm} changes in an external magnetic field B oriented perpendicular to the surface (Supplementary Fig. 3a). τ_{harm} has a maximum at $B = 0.5\,\mathrm{T}$ for the particular tip used within that measurement, indicating a tip effective field of $B_{\text{tip}} = -0.5\,\mathrm{T}$, which compensates an external magnetic field of $B = 0.5\,\mathrm{T}$ to zero. The lifetimes can also be averaged using the geometric mean $\tau_{\text{geo}} = \frac{1}{2}\sqrt{\bar{\tau}_0\bar{\tau}_1}$. As shown by the analysis of the experimental data of Supplementary Fig. 3a using τ_{geo}, the geometric mean is independent of B, and thus not affected by a tip-effective magnetic field.

The analysis of the data of Fig. 3a from the main manuscript using τ_{geo} instead of τ_{harm} is shown in Supplementary Fig. 3b. It reveals a quantitatively identical behavior, which proves

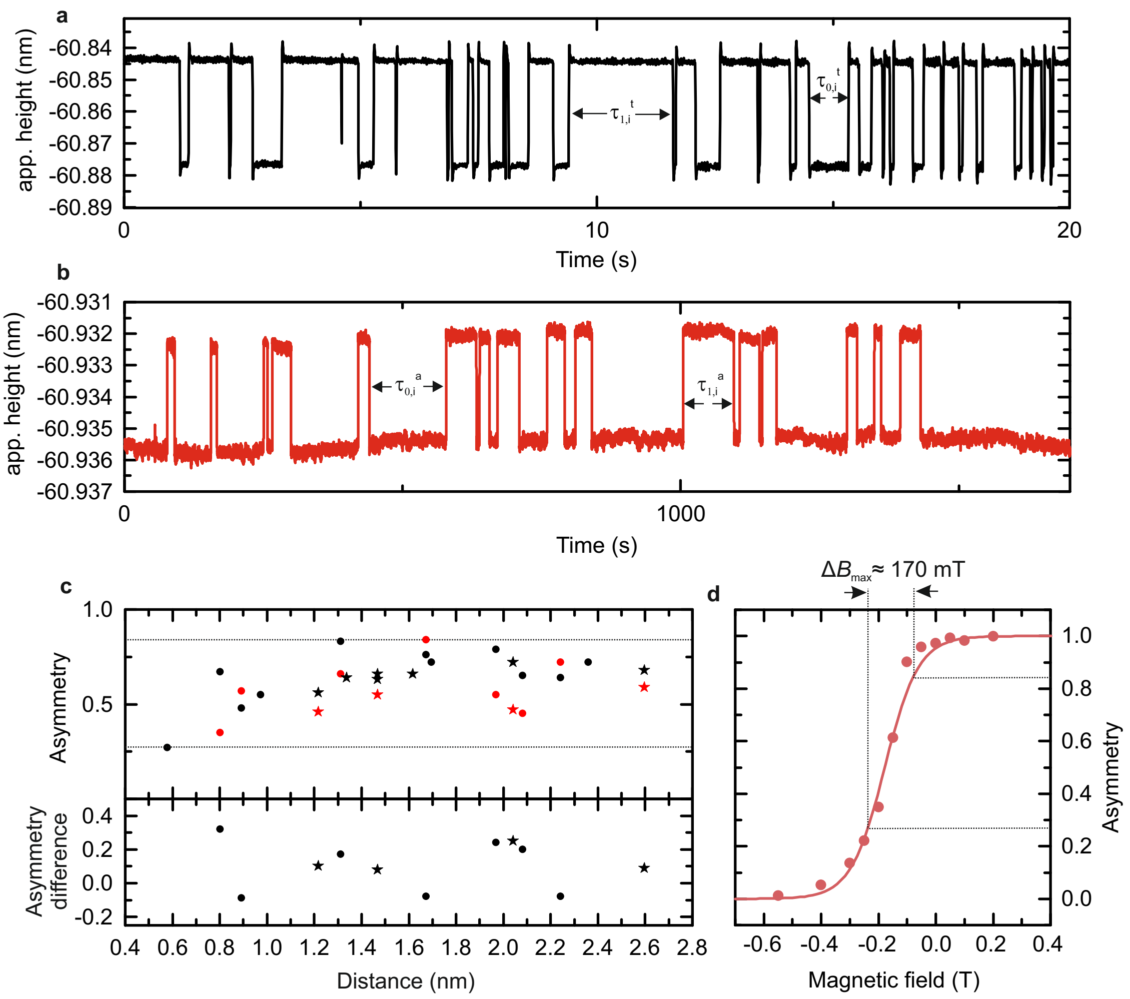

Supplementary Figure 2 | **a** Telegraph signal measured on the trimer in the complex of configuration i (atom-trimer separation $= 2.24\,\text{nm}$). **b** Corresponding telegraph signal measured on the satellite atom in the same complex. **c** Upper panel: Asymmetries for the experimental data shown in Fig. 3a of the main manuscript, determined from the corresponding telegraph signals described in the text (red: on atom, black: on trimer, stars: hcp satellite atom, circles: fcc satellite atoms). For the antiferromagnetically coupled satellite atoms, we plot here $1 - \mathcal{A}$ in order to compare to the values measured on the trimer. The horizontal dashed lines mark the absolute maximum and minimum asymmetry values found in the experiment. Lower panel: difference of the asymmetries measured on trimer and satellite atom in each complex. Measurement parameters of **a** to **c** are $V = -5\,\text{mV}$, $I = 500\,\text{pA}$, $B = 0\,\text{T}$, and $T = 310\,\text{mK}$. **d** Experimentally determined asymmetries in dependence of a magnetic field for an individual trimer without satellite atom (data taken from Fig. 4e of Ref. 2). The absolute maximum and minimum asymmetry values found in the upper panel in **c** are indicated by the vertical lines and result in an upper border of $\Delta B_{\text{max}} \approx 170\,\text{mT}$ for the difference in the tip-effective field acting on the trimer in the two measurement configurations (above trimer, above satellite atom) in each complex for the data shown in Fig. 3a of the main manuscript.

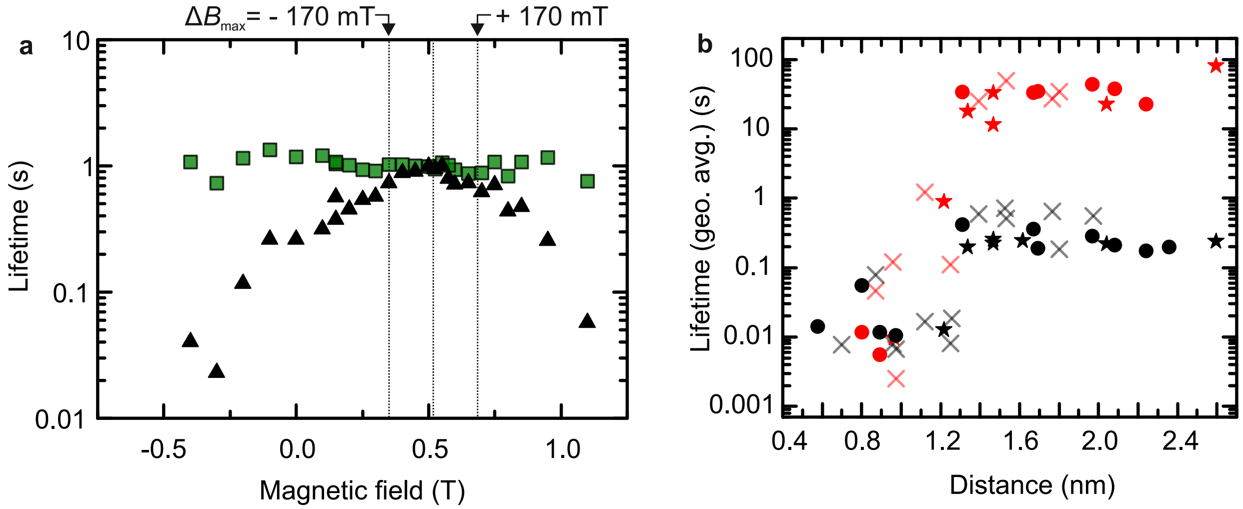

Supplementary Figure 3 | a Mean lifetimes τ_{harm} (black) and τ_{geo} (green) of an individual trimer without satellite atom in dependence of a magnetic field B applied perpendicular to the surface ($V = 5\,\text{mV}$, $I = 500\,\text{pA}$, $T = 310\,\text{mK}$). The vertical lines indicate the upper border of the changes in the tip effective field $\Delta B_{\text{max}} \approx 170\,\text{mT}$ for the measurements of Fig. 3a from the main manuscript. **b** Circles and stars: mean lifetimes τ_{geo} of the experimental data shown in Fig. 3a of the main manuscript (circles: fcc satellite atoms, stars: hcp satellite atoms). Crosses: mean lifetimes τ_{geo} extracted from the measurements on another set of manipulated complexes using a tip different from the one used for Fig. 3a of the main manuscript. Black data points are measured on the trimer and red data points are measured on the satellite atom in each complex. Measurement parameters are the same as in Fig. 3a of the main manuscript.

that the tip effective field can be neglected for the whole distance regime of Fig. 3a of the main manuscript.

Supplementary Fig. 3b additionally contains the geometric means of the lifetimes measured on another set of manipulated complexes using a tip different from the one used for Fig. 3a of the main manuscript. It is extremely improbable that this second tip has the same effective field as the tip used for the measurement of the main manuscript. Nevertheless, the second dataset shows quantitatively the same behavior which again strongly indicates that the tip-effective field can be neglected.

Supplementary Note 4 | Density functional theory calculations

We performed density functional theory (DFT) calculations using the full-potential Korringa-Kohn-Rostoker (KKR) method in a real-space approach, with spin-orbit coupling added self-consistently to the scalar-relativistic approximation[3]. Exchange and correlation effects are treated in the local spin density approximation (LSDA) as parametrized by Vosko, Wilk and Nusair[4].

Supplementary Table 1 | Magnetic properties of the Fe trimer and the Fe adatom deposited on the Pt(111) surface. Both structures are relaxed towards the surface by $20\,\%$ of the interlayer distance. m_s^Fe is the spin moment per Fe atom and m_s^Pt is the total spin moment induced in the Pt surface. K is the uniaxial magnetic anisotropy in the classical Heisenberg model. The experimental values are taken from the literature[1,2].

	fcc-top trimer	fcc adatom
$m_\mathrm{s}^\mathrm{Fe}[\mu_\mathrm{B}]$	3.22	3.41
$m_\mathrm{s}^\mathrm{Pt}[\mu_\mathrm{B}]$	2.02	1.03
K	$-0.19\,\mathrm{meV}$	$-1.92\,\mathrm{meV}$
K (inelastic tunneling spectroscopy)	$-3.22\,\mathrm{meV}$	$-1.66\,\mathrm{meV}$

The pristine Pt(111) surface is modeled by 40 layers of Pt using the experimental lattice constant, $3.92\,\text{Å}$, enclosed by two vacuum regions, each $9.06\,\text{Å}$ thick, with open boundary conditions in the stacking direction. We use 150×150 k-points in the two-dimensional Brillouin zone, and the angular momentum expansions for the scattering wavefunctions are carried out up to $\ell_\mathrm{max} = 3$. The Fe nanostructures are embedded on the Pt surface by defining a real space cluster containing the Fe nanostructure, the surrounding vacuum sites, as well as a hemispherical cluster of Pt atoms containing 15 Pt atoms and 43 Pt atoms for the adatom and the trimer, respectively. This cluster is then self-consistently embedded in the pristine Pt surface. This is repeated for all the possible fcc-stacked positions of the Fe adatom. The magnetic exchange constants are obtained using the infinitesimal rotation method[5,6] and mapped to a classical generalized Heisenberg model $\mathcal{H}_\mathrm{ex} = \frac{1}{2}\sum_{ij} \mathbf{e}_i \underline{\mathbf{J}}_{ij} \mathbf{e}_j$, where i and j sum over all the magnetic atoms in the cluster, with the respective orientations given by the unit vectors $\mathbf{e}_i$ and $\mathbf{e}_j$, and $\underline{\mathbf{J}}_{ij}$ is the exchange tensor (a matrix defining all possible bilinear pairwise interactions). The magnetic anisotropy in the uniaxial form $\mathcal{H}_\mathrm{MAE} = \sum_i K_i e_z^2$ is obtained from band energy differences, as justified by the magnetic force theorem[5], using large hemispherical clusters containing 169 Pt atoms and 149 Pt atoms for the adatom and the trimer, respectively.

Structural relaxation We are not considering any structural relaxations in the Pt surface layer of the pristine surface. However, the nanostructures have a substantial relaxation towards the surface compared to the bulk Pt interlayer distance. The fcc-top Fe trimer has a relaxation of $\sim 17.5\,\%$ [2], whereas the Fe adatom relaxes more, $\sim 26\,\%$ [7]. It was also shown that the central Pt atom below the trimer shifts towards the bulk by $\sim 7.5\,\%$ of the interlayer distance, which had an influence especially on the magnetic anisotropy values. For the studies of the exchange interaction, we have to combine the adatom and the trimer for different positions of the adatom in a common geometry [2]. As a compromise, we set the relaxation to $20\,\%$ of the interlayer distance, and neglect the relaxation of the Pt atom underneath the trimer.

Spin moments and magnetic anisotropy Supplementary Table 1 shows the spin moments of the fcc-top Fe trimer and the Fe adatom separated in the spin moments from the Fe atoms and the spin moments induced in the Pt surface, as well as the magnetic anisotropy energy obtained from collinear DFT calculations and from experiments in the literature[1,2]. The MAE of the adatom agrees reasonably well between theory and experiment. However, the MAE of the trimer obtained from theory is more than one order of magnitude smaller than the experimental value, which is among other things due to the missing structural relaxation of the central Pt atom below the trimer and the neglect of the non-collinearities in the trimer[2].

Vacuum spin polarization Supplementary Fig. 4 shows the energy-dependent spin polarization calculated for three different vertical distances above the fcc Fe adatom and the fcc-top Fe trimer. The spin polarization is defined via the spin-dependent density of states, $\rho^{\uparrow/\downarrow}(E)$, by $(\rho^{\uparrow}(E) - \rho^{\downarrow}(E))/(\rho^{\uparrow}(E) + \rho^{\downarrow}(E))$. Obviously, the spin-polarization in the vacuum above the atom and the trimer at a distance which is comparable to the tip-sample separation has the same sign.

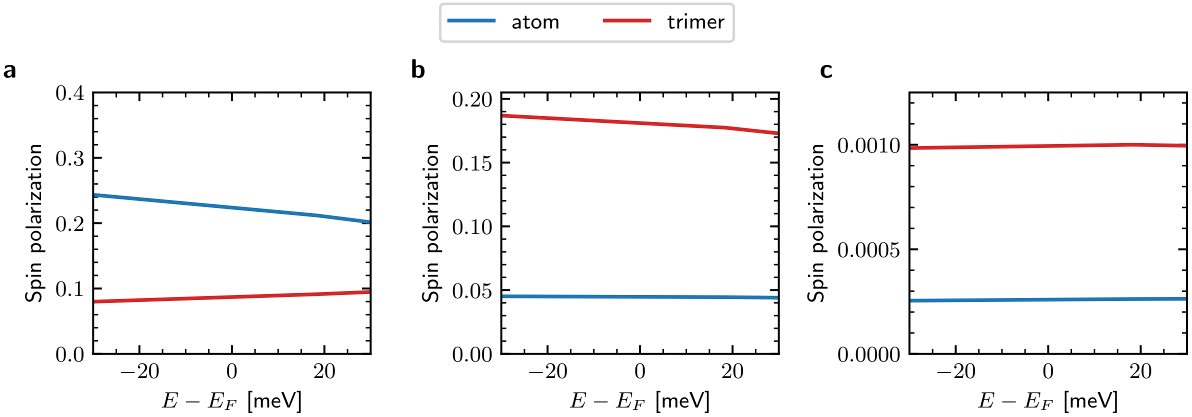

Supplementary Figure 4 | Energy-dependent spin polarization in the vacuum above the fcc Fe adatom (red) and the fcc-top Fe trimer (blue) for three different vertical distances **a** of $2.3\,\text{Å}$, **b** of $4.5\,\text{Å}$ and **c** of $6.8\,\text{Å}$.

Supplementary Note 5 | Ground state spin configuration

The ground state spin configuration of the trimer-adatom nanostructure is obtained from a generalized classical Heisenberg model,

$$\mathcal{H} =\frac{1}{2}\sum_{i\neq j} \mathbf{e}_i \cdot \underline{\mathbf{J}}_{ij}^{\text{classical}} \cdot \mathbf{e}_j + \sum_i K_i \left(e_i^z\right)^2 \tag{2}$$

$$\mathcal{H} =\underbrace{\frac{1}{2}\sum_{i\neq j} J_{ij}\mathbf{e}_i \cdot \mathbf{e}_j}_{\text{isotropic exchange}} + \underbrace{\frac{1}{2}\sum_{i\neq j} \mathbf{D}_{ij}\cdot(\mathbf{e}_i\times\mathbf{e}_j)}_{\text{Dzyaloshinskii-Moriya interaction}} + \underbrace{\frac{1}{2}\sum_{i\neq j} \mathbf{e}_i \cdot \underline{\mathbf{J}}_{ij}^{\text{aniso}} \cdot \mathbf{e}_j}_{\text{anisotropic exchange}} + \underbrace{\sum_i K_i \left(e_i^z\right)^2}_{\text{magnetic anisotropy}} \quad , \tag{3}$$

which sums over all atoms i and j in the cluster containing the trimer and adatom Fe atoms as well as the Pt atoms. The tensor of exchange interactions $\underline{\mathbf{J}}_{ij}^{\text{classical}}$ was here split into its constituents: the isotropic exchange interaction J_{ij}, the Dzyaloshinskii-Moriya interaction $\mathbf{D}_{ij}$, the symmetric anisotropic exchange interaction $\underline{\mathbf{J}}_{ij}^{\text{aniso}} = (\underline{\mathbf{J}}_{ij}^{\text{classical}} + (\underline{\mathbf{J}}_{ij}^{\text{classical}})^{\text{T}})/2 - J_{ij}$, and the uniaxial magnetic anisotropy K_i. The exchange parameters are extracted from the first-principles calculations, whereas the uniaxial anisotropies are taken from previous studies[1,2]. To obtain a simplified Hamiltonian involving only the magnetic Fe atoms, the contributions from the surrounding Pt atoms are absorbed in the exchange interactions between the Fe atoms by employing a scheme described in literature[8]. As the trimer is only mildly noncollinear[2], we assume it to be ferromagnetic, and due to its high magnetic anisotropy we fix its magnetic moment to point perpendicular to the surface plane. The magnetic moment of the adatom is not constrained in its orientation. Summing over the individual magnetic moments of the trimer we obtain an effective Hamiltonian between the trimer and the adatom,

$$\mathcal{H} = \sum_{j\in\text{Trimer}} \mathbf{e}_{\text{ad}} \cdot \underline{\mathbf{J}}_{\text{ad},j}^{\text{classical}} \cdot \mathbf{e}_j + K_{\text{ad}} \left(e_{\text{ad}}^z\right)^2 = \mathbf{e}_{\text{ad}} \cdot \underline{\mathbf{J}}_{\text{ad,tri}}^{\text{classical}} \cdot \mathbf{e}_{\text{tri}} + K_{\text{ad}} \left(e_{\text{ad}}^z\right)^2 \tag{4}$$

$$= \sin\vartheta\cos\varphi\, J_{\text{ad,tri}}^{xz} + \sin\vartheta\sin\varphi\, J_{\text{ad,tri}}^{yz} + \cos\vartheta\, J_{\text{ad,tri}}^{zz} + (\cos\vartheta)^2 K_{\text{ad}} \quad , \tag{5}$$

with the orientations of the trimer magnetic moment $\mathbf{e}_{\text{tri}}$ and of the magnetic moment of the adatom $\mathbf{e}_{\text{ad}}$ described by the spherical angles ϑ and φ with respect to the z-axis which is perpendicular to the sample surface, as indicated in Supplementary Fig. 5. The ground state spin configuration of the adatom can be determined by minimizing the full Hamiltonian (effective exchange between trimer and adatom and uniaxial anisotropy for the trimer and for the adatom) with respect to the orientation of the magnetic moment of the adatom, i.e. min$\{\mathcal{H}(\vartheta,\varphi)\}$.

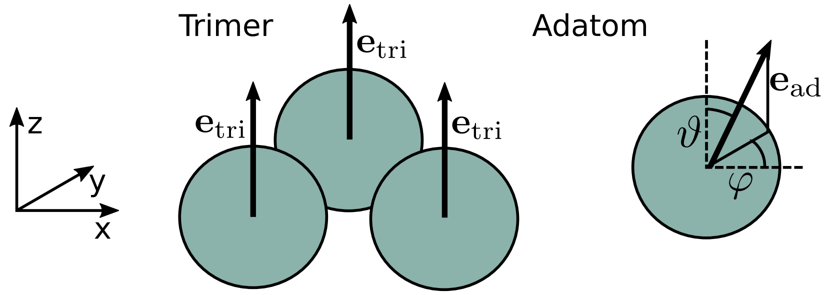

Supplementary Figure 5 | Illustration of the coordinate system used to describe the magnetic moments of the trimer and the adatom. All the magnetic moments of the trimer atoms point perpendicular to the surface in the z-direction, $\mathbf{e}_{\mathrm{tri}}$. The magnetic moment of the adatom points along $\mathbf{e}_{\mathrm{ad}}$ which can be described by the spherical angles ϑ and φ.

Supplementary Note 6 | Master equation model

For the simulation of the telegraph noise lifetimes we utilize a model described in Ref. 9. The total Hamiltonian can be divided into contributions from the tip $\mathcal{H}_T$, the surface $\mathcal{H}_S$, the magnetic nanostructure $\mathcal{H}_{\mathrm{Spin}}$ and a coupling of the nanostructure to the transport electrons $\mathcal{V}$:

$$\mathcal{H} = \mathcal{H}_T + \mathcal{H}_S + \mathcal{H}_{\mathrm{Spin}} + \mathcal{V} \tag{6}$$

The spin Hamiltonian is given by a generalized quantum Heisenberg Hamiltonian with uniaxial magnetic anisotropy:

$$\mathcal{H}_{\mathrm{spin}} = \frac{1}{2}\sum_{ij} \hat{\mathbf{S}}_i \cdot \underline{\mathbf{J}}_{ij} \cdot \hat{\mathbf{S}}_j + \sum_i \mathcal{D}_i \left(\hat{S}_i^z\right)^2 \quad , \tag{7}$$

with the vector spin operator $\hat{\mathbf{S}}_i$ of atom i with the spin quantum number S_i, the full magnetic exchange tensor $\underline{\mathbf{J}}_{ij}$, and the uniaxial anisotropy $\mathcal{D}_i$. The exchange and anisotropy parameters are transformed by replacing the unit vectors from the classical Heisenberg model by the quantum spin vectors yielding

$$\mathcal{D}_i = \frac{K_i}{S_i(S_i+1)} \qquad \text{and} \qquad \underline{\mathbf{J}}_{ij} = \frac{\underline{\mathbf{J}}_{ij}^{\mathrm{classical}}}{\sqrt{S_i(S_i+1)}\sqrt{S_j(S_j+1)}} \quad . \tag{8}$$

The coupling of the nanostructure to the transport electrons is modeled by a Kondo-type Hamiltonian (Appelbaum Hamiltonian[10]) :

$$\mathcal{V} = \sum_{\alpha,\eta,\eta',i} T_i^{\eta,\eta',\alpha} \hat{S}_i^\alpha c_\eta^\dagger \frac{\boldsymbol{\sigma}^\alpha}{2} c_{\eta'} \quad , \tag{9}$$

where α labels the spin-component, η labels the single-particle quantum number of the transport electrons (distinguishing surface or tip), and i labels the trimer or adatom. $T_i^{\eta,\eta',\alpha}$ is the (spin-dependent) exchange-tunneling interaction between the nanostructure and the transport electrons. Assuming only kinetic exchange (which is spin-rotational invariant), one can write

$$T_i^{\eta,\eta',\alpha} = v_i^{\eta} v_i^{\eta'} \mathcal{T}^{\alpha} \quad , \tag{10}$$

where $\mathcal{T}^x = \mathcal{T}^y = \mathcal{T}^z = |\mathcal{T}|$ is isotropic, and $v^{\eta} = v^{\mathrm{S}}, v^{\mathrm{T}}$ are dimensionless factors describing the coupling to the surface and the tip, respectively.
The spin Hamiltonian can be diagonalized yielding the eigenstates $|M\rangle$. Treating the coupling of the spins to the transport electrons via $\mathcal{V}$ as a perturbation, the spin dynamics of the nanostructure can be modeled by using a master equation approach, which connects the diagonal elements of the density matrix, P_M, and the transition matrix, $W_{MM'}$,

$$\frac{\mathrm{d}P_M}{\mathrm{d}t} = \sum_{M'} P_{M'} W_{M'M} - P_M \sum_{M'} W_{MM'} \quad . \tag{11}$$

The transition matrix can be obtained from Fermi's golden rule. In the following, we will restrict ourselves to the inelastic contributions to the transition matrix, since those are the relevant ones for the master equation. The elastic contributions are needed to calculate the full current flowing through the nanostructure,

$$I_{S\to T} = e \sum_{MM'} P_M(V)(W_{MM'}^{S\to T} - W_{MM'}^{T\to S}) \quad . \tag{12}$$

However, by dropping the elastic contributions the model loses two free parameters in exchange for only one experimental information (constant-current). After a few lines of algebra one finds,

$$W_{MM'}^{S\to S} = \mathcal{G}_{MM'}^{SS} k_i \left[\left| \sum_j r_{ij}^{\mathrm{S}} S_{j,MM'}^{+} \right|^2 + \left| \sum_j r_{ij}^{\mathrm{S}} S_{j,MM'}^{-} \right|^2 + 2 \left| \sum_j r_{ij}^{\mathrm{S}} S_{j,MM'}^{z} \right|^2 \right] \quad , \tag{13}$$

$$W_{MM'}^{T\to T} = \mathcal{G}_{MM'}^{TT} k_i r_i^2 \left[|S_{i,MM'}^{+}|^2 \left(1 - \mathcal{P}_{\mathrm{T}}^2\right) + |S_{i,MM'}^{-}|^2 \left(1 - \mathcal{P}_{\mathrm{T}}^2\right) + 2\left(1 + \mathcal{P}_{\mathrm{T}}\right) |S_{i,MM'}^{z}|^2 \right] \quad , \tag{14}$$

$$W_{MM'}^{T\to S} = \mathcal{G}_{MM'}^{TS} k_i r_i \left[|S_{i,MM'}^{+}|^2 \left(1 - \mathcal{P}_{\mathrm{T}}\right) + |S_{i,MM'}^{-}|^2 \left(1 + \mathcal{P}_{\mathrm{T}}\right) + 2|S_{i,MM'}^{z}|^2 \right] \quad , \tag{15}$$

$$W_{MM'}^{S\to T} = \mathcal{G}_{MM'}^{ST} k_i r_i \left[|S_{i,MM'}^{+}|^2 \left(1 + \mathcal{P}_{\mathrm{T}}\right) + |S_{i,MM'}^{-}|^2 \left(1 - \mathcal{P}_{\mathrm{T}}\right) + 2|S_{i,MM'}^{z}|^2 \right] \quad , \tag{16}$$

where $W_{MM'}^{\eta\to\eta'}$ describes the transition of the magnetic nanostructure from state $|M\rangle$ to $|M'\rangle$ due to an electron from η (tip/surface) to η' (tip/surface) when the current flows through atom i. The function $\mathcal{G}_{MM'}^{\eta\eta'}$ is given by

$$\mathcal{G}_{MM'}^{\eta\eta'} = \frac{\Delta_{MM'}^{\eta\eta'}}{1 - \exp\left(-\Delta_{MM'}^{\eta\eta'}/k_{\mathrm{B}}T\right)} \qquad \text{with} \qquad \Delta_{MM'}^{\eta\eta'} = E_M - E_{M'} + \mu_{\eta} - \mu_{\eta'} \quad , \tag{17}$$

where $E_M = \langle M|\mathcal{H}_{\mathrm{spin}}|M\rangle$, k_{B} is the Boltzmann constant, T is the temperature, and μ_{η} is the chemical potential (containing the external voltage). $S_{i,MM'}^{\alpha}$ are the matrix elements of the spin

of atom i, $S^{\alpha}_{i,MM'} = \langle M|\hat{S}^{\alpha}_i|M'\rangle$. $\mathcal{P}_{\mathrm{T}}$ is the spin polarization of the tip, which is set to 10%. The prefactors (which cannot be determined from DFT) are given by,

$$k_i = \frac{2\pi\mathcal{T}^2(v_i^{\mathrm{S}})^4}{\hbar}\frac{\rho_{\mathrm{S}}^2}{4} \quad , \quad r_i = \frac{\rho_{\mathrm{T}}}{\rho_{\mathrm{S}}}\left(\frac{v_i^{\mathrm{T}}}{v_i^{\mathrm{S}}}\right)^2 \quad \text{and} \quad r_{ij}^{\mathrm{S}} = \left(\frac{v_j^{\mathrm{S}}}{v_i^{\mathrm{S}}}\right)^2 \quad , \tag{18}$$

where $\rho_{\mathrm{S(T)}}$ is the density of states of the surface (tip). The tip-surface coupling ratio of the trimer r_{tri} is taken from Ref. 2. The tip coupling of the adatom, $v^{\mathrm{T}}_{\mathrm{ad}}$, is assumed to be the same as the tip coupling of the trimer. The unknown ratio between the surface couplings, $v^{\mathrm{S}}_{\mathrm{ad,tri}}$, is set to 3.5, which yields the best agreement between the experimental data and the simulation. The prefactor k_{tri} is adjusted, such that the lifetime of the isolated trimer fits the experimental data.

Calculation of the lifetimes The master equation can be written in a matrix form as

$$\frac{\mathrm{d}P_M}{\mathrm{d}t} = \sum_{MM'} A_{MM'}P_{M'} \quad \Rightarrow \quad \frac{\mathrm{d}\mathbf{P}}{\mathrm{d}t} = \underline{\mathbf{A}}\mathbf{P} \quad , \tag{19}$$

with $A_{MM'} = W_{M'M} - \delta_{MM'}\sum_{M''}W_{MM''}$. The solution of the ordinary differential equation is given by a matrix exponential, $\mathbf{P}(t) = \exp(\underline{\mathbf{A}}t)\mathbf{P}(t=0)$. The non-hermitian matrix $\underline{\mathbf{A}}$ can be diagonalized by using

$$\underline{\mathbf{D}} = \underline{\mathbf{S}}^{-1}\underline{\mathbf{A}}\underline{\mathbf{S}} \quad \text{with} \quad \underline{\mathbf{S}} = (\mathbf{R}_1, \mathbf{R}_2, \ldots) \quad , \tag{20}$$

$$\underline{\mathbf{S}}^{-1} = \begin{pmatrix} \mathbf{L}_1^{\mathrm{T}} \\ \mathbf{L}_2^{\mathrm{T}} \\ \vdots \end{pmatrix} \quad \text{and} \quad \underline{\mathbf{D}} = \begin{pmatrix} \lambda_1 & 0 & \ldots \\ 0 & \lambda_2 & \ldots \\ \vdots & \vdots & \ddots \end{pmatrix} \quad , \tag{21}$$

where $\mathbf{R}_n$ are the right eigenvectors of A and $\mathbf{L}_n$ are the left eigenvectors fulfilling $A\mathbf{R}_n = \lambda_n\mathbf{R}_n$ and $\mathbf{L}_nA = \lambda_n\mathbf{L}_n$, which yields

$$\mathbf{P}(t) = \underline{\mathbf{S}}\exp(\underline{\mathbf{D}}t)\underline{\mathbf{S}}^{-1}\mathbf{P}(t=0) \quad . \tag{22}$$

From the physical point of view, we know that $\underline{\mathbf{A}}$ should have a stationary solution with eigenvalue 0. Furthermore, the eigenvalues have to be smaller than 0 in order to ensure the normalization of $\mathbf{P}(t)$. All the eigenvalues, λ_n, correspond to different transient states. The particular lifetimes of those states are given by $\tau_n = -1/\lambda_n$. We assume that the longest lifetime corresponds to the full flip of the spin moment, and therefore it relates to the experimentally measured telegraph noise lifetime.

Projection of the lifetimes To obtain separate lifetimes for each spin, trimer and atom, we calculate the probability of finding the system in a subspace involving only the trimer or the atom. The density matrix of the system, which relates to the probability of finding the full spin system in state $|M\rangle$, is given by $\rho(t) = \sum_M P_M(t)|M\rangle\langle M|$. The matrix $A_{MM'}$ given in terms of left and right eigenvectors, $A_{MM'} = \sum_\alpha R^\alpha_M \lambda^\alpha L^\alpha_{M'}$, yields for the probability,

$$P_M(t) = \sum_{\alpha M'} R^\alpha_M \exp(\lambda^\alpha t) L^\alpha_{M'} P_{M'}(0) \quad . \tag{23}$$

Supplementary Table 2 | Simulated lifetimes of the isolated trimer and adatom. For the probed values a voltage of $5\,\mathrm{mV}$ is applied between the tip and the surface leading to a tunneling current, whereas the unprobed ones are obtained considering only interactions between the quantum spin and the surface electrons.

	fcc-top trimer	fcc adatom
probed	$0.465\,\mathrm{s}$	$0.10\,\mathrm{ms}$
unprobed	$8.24 \times 10^5\,\mathrm{s}$	$43.0\,\mathrm{ms}$

The eigenstates of the full spin Hamiltonian can be written in the tensor product basis (atom and trimer) as

$$|M\rangle = \sum_{m_1 m_2} C^M_{m_1 m_2} |m_1\rangle \otimes |m_2\rangle \quad . \tag{24}$$

Thus the probability to find the atom in state $|m_1\rangle$ is given by

$$P_{m_1}(t) = \sum_{m_2} \langle m_1| \otimes \langle m_2|\rho(t)|m_1\rangle \otimes |m_2\rangle \tag{25}$$

$$= \sum_{m_2} \langle m_1| \otimes \langle m_2| \sum_M P_M(t)|M\rangle\langle M||m_1\rangle \otimes |m_2\rangle \tag{26}$$

$$= \sum_{m_2} \langle m_1| \otimes \langle m_2| \sum_{\substack{M \\ m_1' m_2' \\ m_1'' m_2''}} P_M(t) C^M_{m_1' m_2'} |m_1'\rangle \otimes |m_2'\rangle \left(C^M_{m_1'' m_2''}\right)^* \langle m_1''| \otimes \langle m_2''||m_1\rangle \otimes |m_2\rangle \tag{27}$$

$$= \sum_{M m_2} P_M(t) \left|C^M_{m_1 m_2}\right|^2 = \sum_\alpha \exp(\lambda^\alpha t) \underbrace{\sum_{M M' m_2} \left|C^M_{m_1 m_2}\right|^2 R^\alpha_M L^\alpha_{M'} P_{M'}(0)}_{P^\alpha_{m_1}} \quad . \tag{28}$$

The same procedure holds for the probability to find the trimer in state $|m_2\rangle$. The lifetime of the clusters (atom and trimer) is therefore given by the eigenvalue α, which has the highest occupation in $P^\alpha_{m_1/m_2}$ for $|m_1\rangle$ and $|m_2\rangle$ being one of the ground states with maximal z-projection of the atom and the trimer, respectively. As initial configuration $P_M(0)$ we use the thermal equilibrium of the total spin system..

Supplementary Note 7 | Simulated lifetimes

Simulated lifetimes of the isolated clusters The simulated lifetimes of the isolated adatom and isolated trimer are shown in Supplementary Table 2. The adatom and the trimer are modeled

according to the main text as spins with $S = 5/2$ and $S = 11/2$ with a uniaxial anisotropy of $\mathcal{D} = -0.19\,\mathrm{meV}$[1] and $\mathcal{D} = -0.09\,\mathrm{meV}$[2], respectively. Keep in mind that our master equation model contains only thermal excitations. This could lead to inaccurate values for the unprobed lifetimes, since quantum fluctuations[11], e.g. originating from zero-point spin fluctuations, can be important in the limit of vanishing external voltage. If a voltage is applied the spin system is fully excited by the voltage, which makes those fluctuations less relevant.

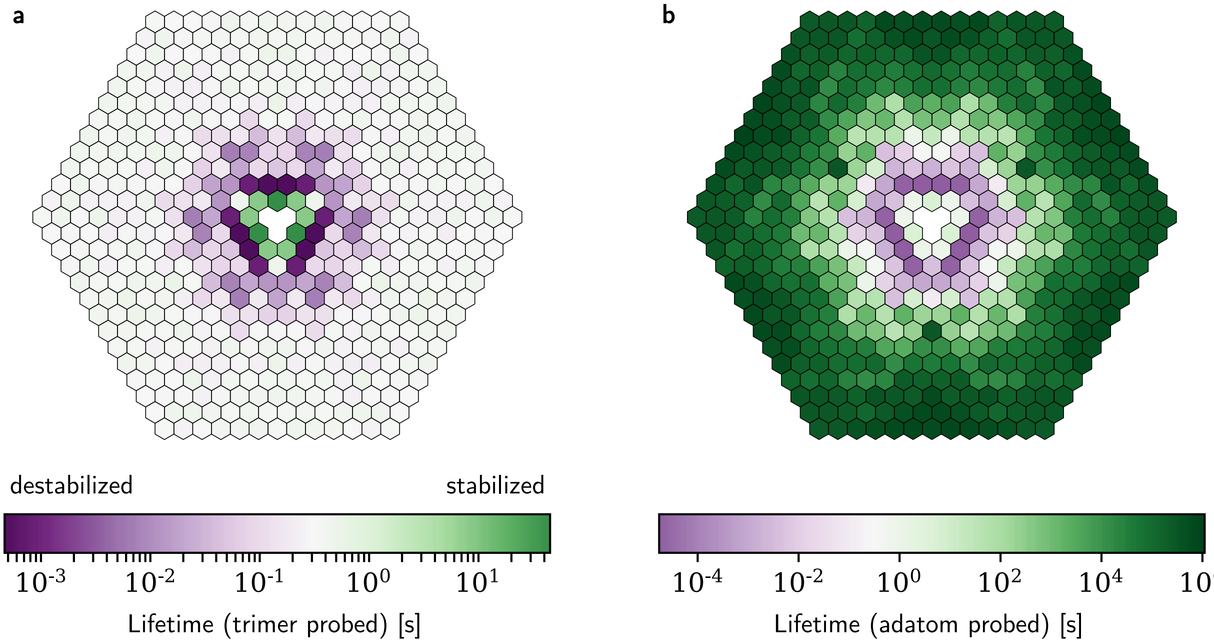

Supplementary Figure 6 | Longest lifetimes of the system for several positions of the adatom obtained from the master equation approach. The colorscale indicates a stabilization (green) and a destabilization (purple) in comparison to the isolated probed trimer. **a** Trimer is probed. **b** Adatom is probed.

Lifetimes for all fcc sites The simulated lifetimes for all the possible fcc-stacked sites of the adatom are shown in Supplementary Fig. 6. Shown are the longest lifetimes of the system, which always relate to the lifetimes of the trimer (see projected lifetimes). For the intermediate regime the trimer is strongly destabilized. Interestingly, when the full system conserves one of the initial mirror planes the trimer is significantly less destabilized. In the long distance regime the lifetime of the isolated trimer is recovered.

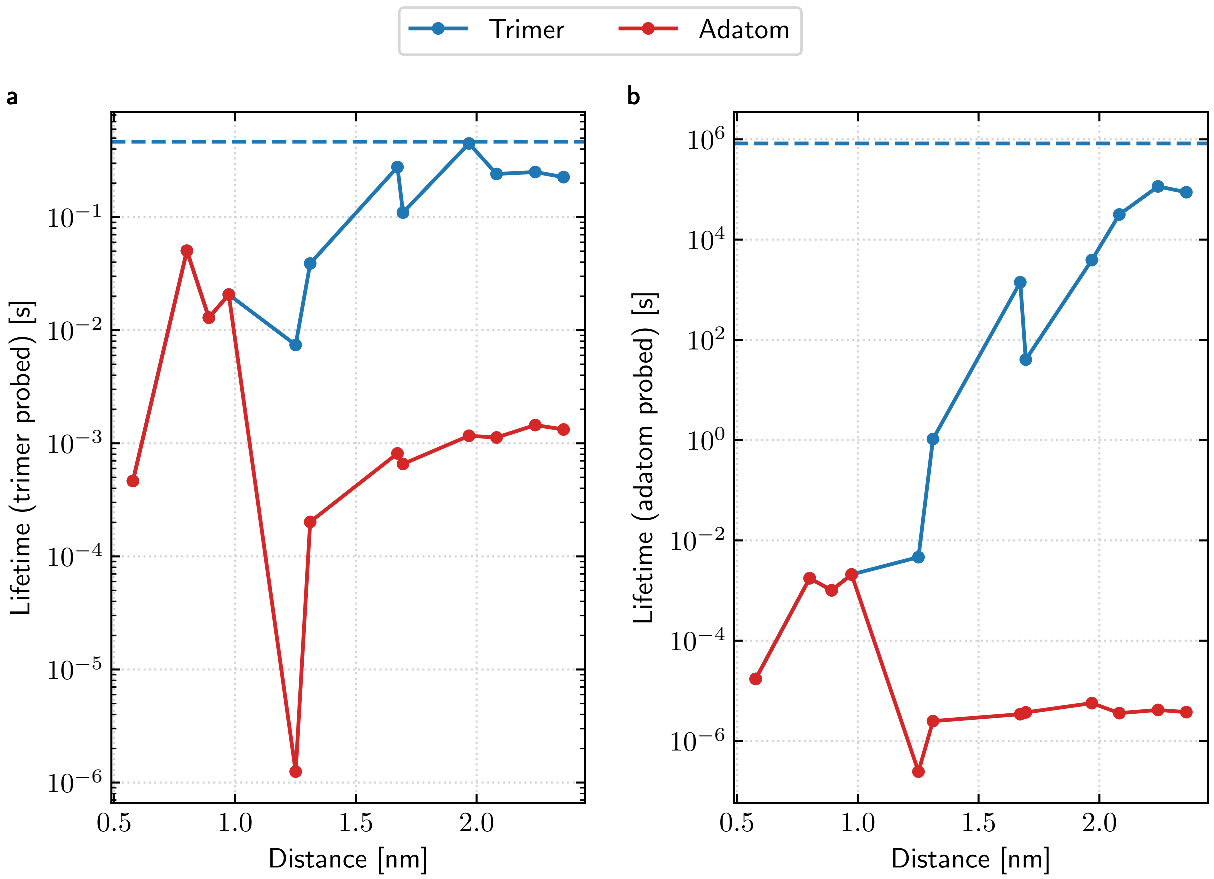

Supplementary Figure 7 | Simulated lifetimes for the experimental positions projected onto the trimer (blue curve) and the adatom (red curve). The blue dashed curve is the simulated lifetime of the isolated probed (a) and unprobed (b) trimer. **a** Trimer is probed. **b** Adatom is probed.

Projected lifetimes The lifetimes projected onto the adatom and trimer for the experimental positions are shown in Supplementary Fig. 7. As mentioned above, the longest lifetime of the system corresponds to processes involving the trimer spin. In the long distance regime, the lifetime of the adatom is orders of magnitude smaller than the lifetime of the trimer. Interestingly, this projection allows us to conclude that, via the experimental contrast mechanism described above in Supplementary Note 1, where the shorter lifetime of the adatom is averaged out, the probed adatom shows the signature of the lifetime of the unprobed trimer.

Dependence of the lifetime on the strength of the exchange interaction To gain more insight on the effects of the different exchange contributions on the lifetimes, we simulated the lifetimes as a function of the exchange interaction strengths. The adatom and the trimer are modeled as quantum spins with uniaxial anisotropy with the values according to the main text.

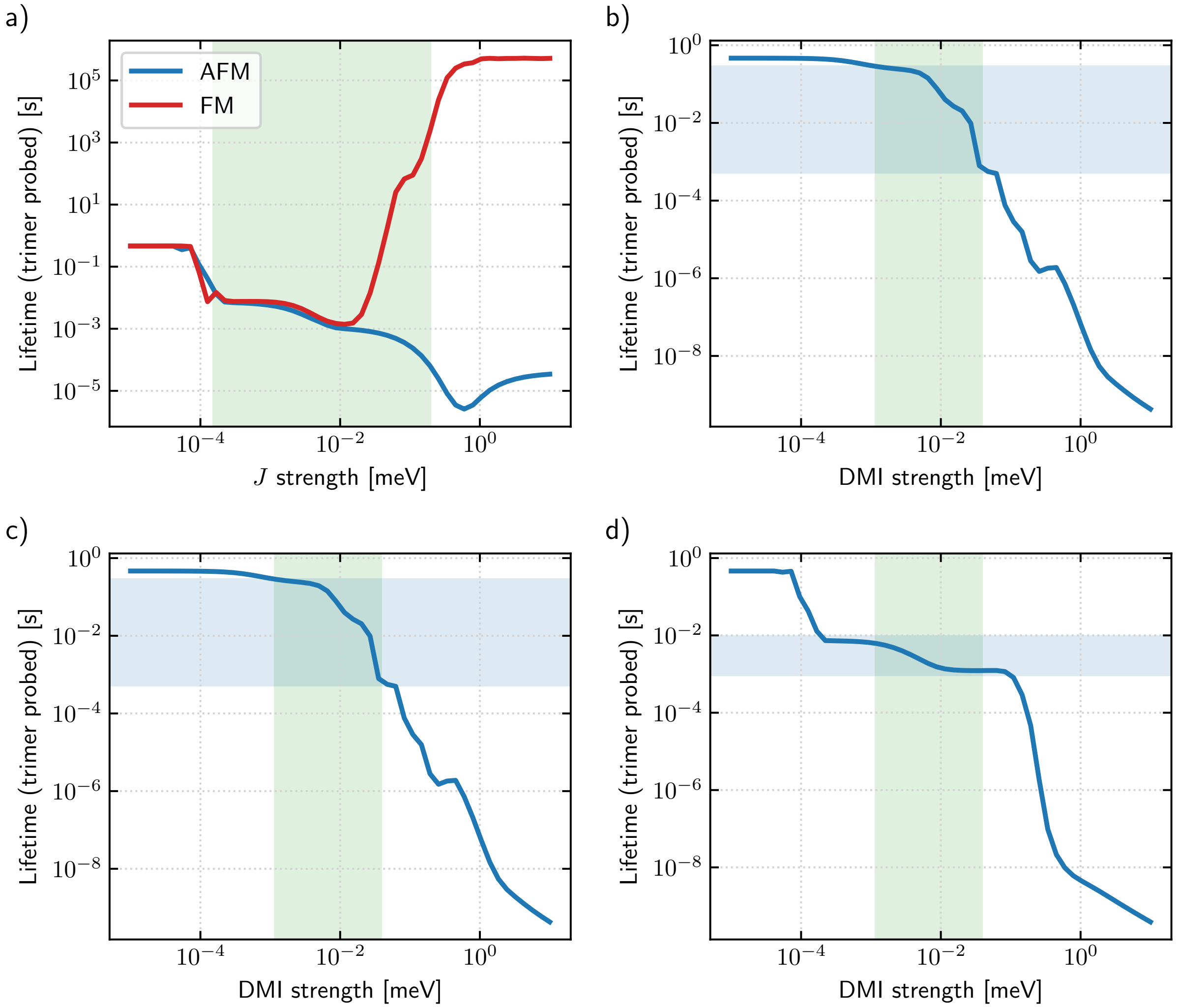

Supplementary Figure 8 | Simulated lifetimes as function of the exchange interaction strength. The trimer and the adatom are modeled by spins with uniaxial anisotropy as described in the main text. The green background indicates the range of the different interactions for the experimental points. **a** Purely isotropic exchange with ferromagnetic (red curve) and antiferromagnetic coupling (blue curve). **b** Purely DMI in the x-direction (D_x). **c** Purely DMI in the y-direction (D_y). **d** Purely DMI in the z-direction (D_z).

In Supplementary Fig. 8a the lifetimes as functions of the strength of the isotropic exchange are shown. When the coupling (FM and AFM) is weak, it destabilizes the trimer by linking it to the less stable spin (the adatom). When the coupling reaches the same order of magnitude as the anisotropy of both constituents, a splitting in the lifetimes obtained for the FM and the AFM couplings occurs. The FM coupling stabilizes the total structure, whereas the AFM coupling destabilizes it.

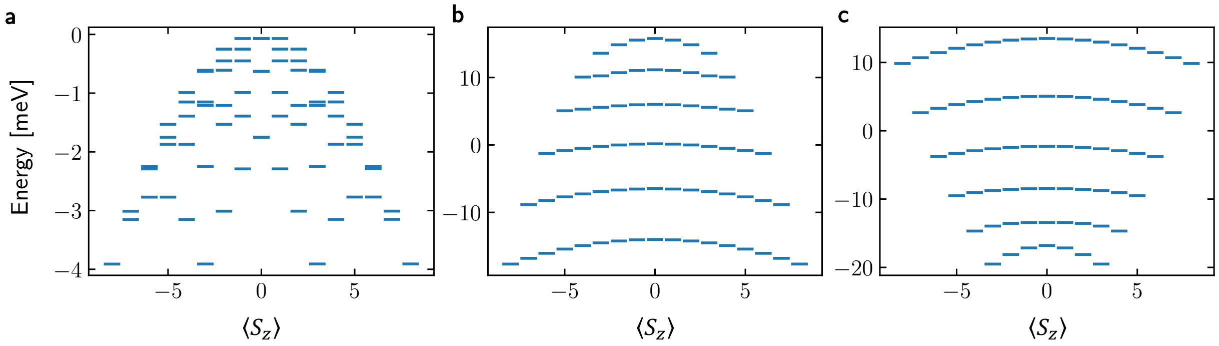

Supplementary Figure 9 | $\langle S_z \rangle$-projected eigenstates of the spin Hamiltonian of the coupled trimer and adatom. **a** Uncoupled ($J = 0\,\mathrm{meV}$). **b** FM coupled ($J = -1.0\,\mathrm{meV}$). **c** AFM coupled ($J = 1.0\,\mathrm{meV}$).

A simple explanation for this can be given by the eigenstates of the system shown in Supplementary Fig. 9. For the uncoupled system (a) the combined parabolas of the small and large spin are obtained. For strong FM and AFM coupling (b and c) the energy levels are split in subspaces related to the total spin of the combined system. For FM coupling the ground state is in the large subspace of maximum total spin, whereas for AFM coupling the ground state belongs to the smaller subspace of the lowest total spin. This results in a stabilization for FM coupling and a destabilization for AFM coupling.

The effects of the different components of the DM interaction are shown in Supplementary Fig. 8b-d. The DM interaction leads to a mixing of the eigenstates, which in turn lowers the lifetimes. Especially the x and y components lead to a drastic decrease of the lifetime in the range of the DMI strength found for the experimental positions.

Influence of the symmetry on the lifetime To analyze the symmetry aspect for the trimer surrounded by three additional satellite atoms, we use the master equation model to simulate the lifetime of the four coupled spins. In a first step, the adatoms are placed along the high symmetry directions as illustrated in Figure 4d in the main text. The exchange interactions for this particular configuration are extracted from density functional theory and the resulting lifetime $\tau(0°)$ is calculated as a reference. In a second step, the exchange tensor between the trimer and one of the satellite atoms is rotated in $5°$ steps as illustrated in Supplementary Figure 10a. This ensures that the strengths of the interactions are kept, while the symmetry of the system as a whole changes. The calculated lifetimes which are shown in Supplementary Figure 10b show an exponential decrease of the lifetimes as a function of the rotation angle. By rotating the exchange tensor out of the high symmetry direction the symmetric anisotropic part of the exchange interaction induces an effective non-local magnetic anisotropy, which in turn lowers the stability of the trimer by orders of magnitude. Keep in mind that the local properties of the trimer such as anisotropies and internal exchange interactions are not changed in this study. Therefore, the simulations of Figure 10a prove that merely the symmetry is essential for getting a large spin lifetime.

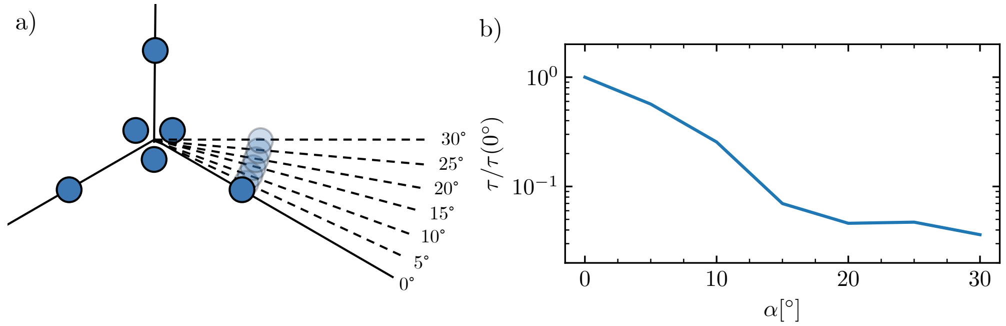

Supplementary Figure 10 | Dependence of the lifetime of the complex on the symmetry of the arrangement of the satellite atoms. For these calculations, the computed RKKY interactions between the trimer and each adatom (isotropic exchange, DMI, anisotropic exchange) were kept fixed, in order to investigate the influence of merely the symmetry on the spin dynamics. One of the satellite atoms was then displaced from the high-symmetry orientation, see panel (a), and its exchange tensor with the trimer was transformed by the respective rotation matrix. This ensures that the strength and other properties of the interactions are kept. The lifetime of the trimer is given in panel (b), which shows that the lifetime drops exponentially as the misalignment increases.

Supplementary Note 8 | Distance dependence of the magnetic exchange constants

The exchange constants for all fcc-stacking sites of the adatom are shown in Supplementary Fig. 11. The isotropic exchange, J, is shown in (a). The absolute value of the DM vector, $|\mathbf{D}_{ij}| = \sqrt{D_\mathrm{x}^2 + D_\mathrm{y}^2 + D_\mathrm{z}^2}$, is shown in (b). To characterize the anisotopic exchange, $\underline{\mathbf{J}}_\mathrm{aniso}$, we diagonalized this part of the exchange tensor and took the maximal difference between the eigenvalues, which is shown in (c).

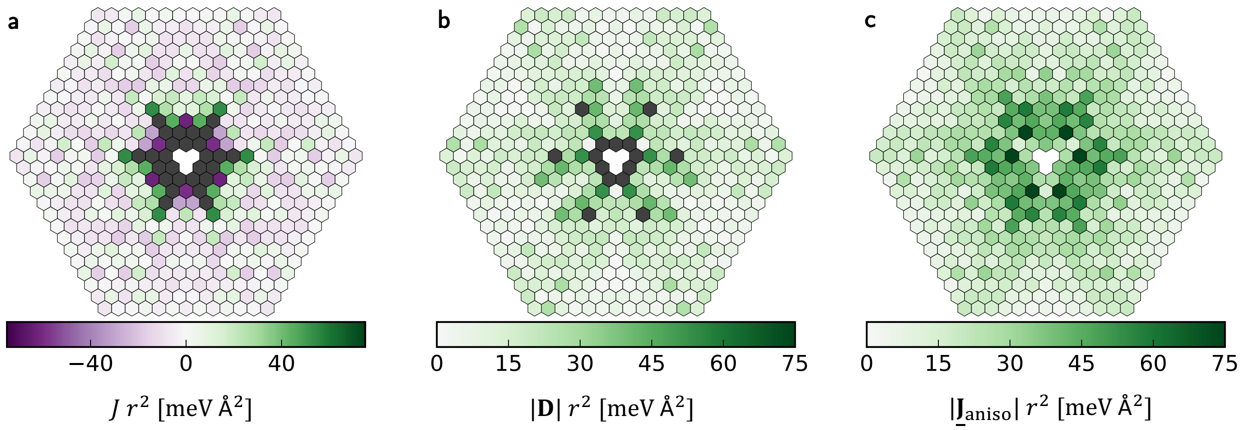

Supplementary Figure 11 | Calculated magnetic exchange interactions for all fcc-stacked sites of the adatom as extracted by mapping DFT to the classical Heisenberg model. The values are scaled by the square of the distance between the adatom and the trimer. To better compare the different contributions the range of all color scales are the same. Values exceeding the color scale are colored grey. **a** Isotropic exchange. **b** Dzyaloshinskii-Moriya interaction. Shown is the absolute value of the Dzyaloshinskii-Moriya vector. **c** Symmetric anisotropic exchange. Shown is the maximal difference between the eigenvalues of the anisotropic exchange tensor.

Supplementary References

1. Khajetoorians, A. A. *et al.* Spin Excitations of Individual Fe Atoms on Pt(111): Impact of the Site-Dependent Giant Substrate Polarization. *Physical Review Letters* **111**, 157204 (2013).

2. Hermenau, J. *et al.* A gateway towards non-collinear spin processing using three-atom magnets with strong substrate coupling. *Nature Communications* **8**, 642 (2017).

3. Bauer, D. S. G. *Development of a Relativistic Full-Potential First-Principles Multiple Scattering Green Function Method Applied to Complex Magnetic Textrues of Nano Structures at Surfaces*. Phd Thesis, RWTH Aachen University (2013).

4. Vosko, S. H., Wilk, L. & Nusair, M. Accurate spin-dependent electron liquid correlation energies for local spin density calculations: A critical analysis. *Canadian Journal of physics* **58**, 1200–1211 (1980).

5. Lichtenstein, A. I., Katsnelson, M. I., Antropov, V. P. & Gubanov, V. A. Local spin density functional approach to the theory of exchange interactions in ferromagnetic metals and alloys. *Journal of Magnetism and Magnetic Materials* **67**, 65–74 (1987).

6. Mankovsky, S. *et al.* Effects of spin-orbit coupling on the spin structure of deposited transition-metal clusters. *Physical Review B* **80**, 014422 (2009).

7. Błoński, P. & Hafner, J. Density-functional theory of the magnetic anisotropy of nanostructures: an assessment of different approximations. *Journal of Physics: Condensed Matter* **21**, 426001 (2009). URL http://stacks.iop.org/0953-8984/21/i=42/a=426001.

8. Polesya, S. *et al.* Finite-temperature magnetism of Fe_xPd_{1-x} and Co_xPt_{1-x} alloys. *Physical Review B* **82**, 214409 (2010).

9. Delgado, F. & Fernández-Rossier, J. Spin dynamics of current-driven single magnetic adatoms and molecules. *Physical Review B* **82**, 134414 (2010).

10. Appelbaum, J. s-d Exchange Model of Zero-Bias Tunneling Anomalies. *Physical Review Letters* **17**, 91–95 (1966).

11. Ibañez-Azpiroz, J., dos Santos Dias, M., Blügel, S. & Lounis, S. Zero-point spin-fluctuations of single adatoms. *Nano Letters* **16**, 4305–4311 (2016). URL https://doi.org/10.1021/acs.nanolett.6b01344. PMID: 27248465, https://doi.org/10.1021/acs.nanolett.6b01344.
